# Association between agonal breathing and outcomes after out-of-hospital cardiac arrest: a retrospective study

**DOI:** 10.1016/j.acepjo.2026.100345

**Published:** 2026-03-07

**Authors:** Hiroaki Taniguchi, Kohei Yamada, Shinnosuke Kitano, Naofumi Bunya, Yosuke Homma, Takashi Tagami, Makoto Aoki, SOS-KANTO 2017 Study Group

**Affiliations:** 1 Department of Emergency Medicine, Japan Self Defense Forces Sapporo Hospital, Sapporo, Japan; 2 Department of Traumatology and Critical Care Medicine, National Defense Medical College Hospital, Tokorozawa, Japan; 3 Department of Emergency and Disaster Medicine, The Jikei University School of Medicine, Tokyo, Japan; 4 Department of Emergency Medicine, Sapporo Medical University, Sapporo, Japan; 5 Department of Emergency and Critical Care Medicine, Chiba Kaihin Municipal Hospital, Chiba, Japan; 6 Division of Traumatology, National Defense Medical College Research Institute, Tokorozawa, Japan

**Keywords:** agonal breathing, gasping, signs of life, out-of-hospital cardiac arrest

## Abstract

**Objectives:**

Out-of-hospital cardiac arrest (OHCA) in patients remains associated with poor survival and neurologic outcomes. Agonal breathing is associated with improved outcomes but lacks a standardized definition or timing for assessment. The aim of this study was to examine the association between agonal breathing and favorable neurologic outcome in patient with OHCA, focusing on its pattern at emergency medical services (EMS) contact and hospital arrival.

**Methods:**

This prospective cohort study collected data from patients with OHCA in Japan between 2019 and 2021, using the SOS-KANTO registry. Agonal breathing was assessed at EMS arrival and hospital arrival and categorized into 3 groups: no agonal breathing, prehospital or hospital agonal breathing, and prehospital and hospital agonal breathing. The primary outcome was favorable neurologic outcome (cerebral performance category of 1 or 2). Multivariable logistic regression was performed to examine the association between agonal breathing and outcomes.

**Results:**

After multiple imputation for missing data, 9909 patients with OHCA were categorized: no agonal breathing (n = 8956, 90.4%), prehospital or hospital agonal breathing (n = 860, 8.7%), and prehospital and hospital agonal breathing (n = 93, 0.9%). Favorable neurologic outcomes were observed in 1.8%, 13.0%, and 19.4% of patients, respectively. Compared with patients with no agonal breathing, patients with prehospital or hospital agonal breathing had higher odds of favorable neurologic outcome (odds ratio, 2.91 [95% CI, 2.08–4.08]), as did patients with prehospital and hospital agonal breathing (odds ratio, 4.63 [95% CI, 2.26–9.49]).

**Conclusion:**

Agonal breathing was associated with higher odds of a favorable neurologic outcome in patients after OHCA, particularly when observed both at EMS contact and on hospital arrival.

**The Bottom Line**

Agonal breathing has been associated with better outcomes in cardiac arrest, but the clinical significance of its timing has remained uncertain. Using a large regional registry of nearly ten thousand patients, we compared patients who showed agonal breathing at neither, one, or both of two key moments: arrival of emergency medical services and arrival at the hospital. Patients who exhibited agonal breathing at both times showed the highest likelihood of a favorable outcome. These findings suggest that the temporal pattern of agonal breathing may serve as a simple bedside indicator of meaningful recovery potential.

## 1 Introduction

### 1.1 Background

Out-of-hospital cardiac arrest (OHCA) is a globally important issue. Although outcomes of OHCA have improved, recent data show that the results are still poor, with a 30-day survival rate of 10.7%^1^ and a favorable neurologic outcome rate of 5.7%.^2^ To improve outcomes, it is essential to properly allocate resources to patients who may benefit from hospital treatment, considering the prognostic factors. Identifying prognostic factors associated with clinical outcomes is crucial.^3^^,^^4^ Factors such as witnessed arrest, bystander cardiopulmonary resuscitation (CPR), initial shockable rhythm, and return of spontaneous circulation (ROSC) are associated with favorable outcomes, but predicting outcomes remains challenging even after incorporating these factors.^3^^,^^4^

### 1.2 Importance

Agonal breathing, also known as gasping, is an irregular and deep respiratory movement observed during CPR, indicating the presence of residual brainstem function. Prior literature has suggested that agonal breathing may be associated with increased favorable neurologic outcomes.^5^^,^^6^ A recent meta-analysis reported that agonal breathing is associated with favorable neurologic outcomes.^7^

Agonal breathing may be a useful prognostic factor, but there is no consensus on its measurement. Studies have assessed agonal breathing at various stages: during the emergency call,^5^^,^^8^^,^^9^ at emergency medical services (EMS) arrival,^6^^,^^10^^,^^11^ at hospital arrival,^12^^,^^13^ and after endotracheal intubation.^14^ However, most previous studies have examined agonal breathing at only one time point, but agonal breathing may be present at later points of resuscitation.

### 1.3 Goals of This Investigation

We aimed to describe the association between agonal breathing and outcomes after OHCA. We hypothesized that the presence of agonal breathing at both EMS arrival and hospital arrival would be associated with higher odds of favorable neurologic outcomes compared with isolated or no agonal breathing.

## 2 Methods

### 2.1 Study Design and Settings

This study is a retrospective analysis of a prospective multicenter cohort study using data from the SOS-KANTO registry, conducted in the Kanto region of Japan. The region, encompassing the capital and 6 surrounding prefectures, covers approximately 32,423 km^2^ with a population of about 43.65 million and includes both densely populated urban areas and less populated rural regions. The registry comprises patients transported to 42 emergency hospitals between September 2019 and March 2021.^15^

All participating institutions obtained approval from their respective institutional review boards, and informed consent was waived. The registry collected data using an opt-out approach. Participating hospitals made information about data collection and ethical approval publicly available on their websites.

This study protocol was approved by the Ethics Committee of the National Defense Medical College, and the approval number for this study is 5140. The research adhered to the guidelines for clinical trials involving pharmaceuticals and the Declaration of Helsinki. This study followed the Strengthening the Reporting of Observational Studies in Epidemiology (STROBE) guidelines.

### 2.2 Selection of Participants

In this study, we included all patients with OHCA who were transported by EMS. OHCA was defined as the cessation of mechanical heart activity, confirmed by the absence of circulatory signs, including the lack of a palpable pulse and unresponsiveness. The study aimed to include as many patients as possible to maximize the statistical power of the analysis. Because agonal breathing may have significance in all patient subsets, we included all patient subgroups, including children and traumatic arrests.^16^^,^^17^ Patients who were not transported to hospitals were not included in this registry, as EMS in Japan generally transport all patients with OHCA unless obvious death criteria are met, and neurologic outcomes cannot be assessed for nontransported cases.^18^^,^^19^ As this was an observational study using an existing data set, no formal sample size calculation was performed.

### 2.3 Measurements

Data were prospectively collected in accordance with the Utstein Registry format, including both prehospital and in-hospital data. Prehospital data were collected by EMS and included patient demographic characteristics, pre-existing health conditions, comorbidities, whether the event was witnessed, bystander CPR, initial rhythm at the scene, time intervals to hospital arrival, interventions such as advanced airway management (bag-valve-mask ventilation, supraglottic airway device, or endotracheal intubation) and adrenaline administration, ROSC before hospital arrival, and prehospital physician intervention. Age was modeled as a continuous variable, and other covariates were specified according to their original measurement scales.

In-hospital data comprised initial laboratory results, medications, and treatments, including ROSC after hospital arrival, electrocardiogram patterns, defibrillation, endotracheal intubation, extracorporeal cardiopulmonary resuscitation (ECPR), intra-aortic balloon pump, coronary angiography, and renal replacement therapy. Additionally, data on the cause of cardiac arrest (cardiogenic or noncardiogenic), length of hospital stay, and neurologic status at 30 days posthospitalization were collected.

### 2.4 Primary Exposure - Agonal Breathing

The primary exposure was the presence of agonal breathing during resuscitation. In this study, agonal breathing was defined as irregular, deep, or gasping respirations suggestive of residual brainstem activity during cardiac arrest,^20^ which is widely recognized in clinical practice and resuscitation training in Japan. Agonal breathing was assessed by EMS personnel in the prehospital setting and by physicians upon hospital arrival, and was recorded separately at both time points. Recognition of agonal breathing was based on direct observation of gasping or irregular respirations rather than on objective monitoring and was conducted during resuscitation regardless of airway management. For the analysis, we characterized agonal breathing in 2 ways: the presence of agonal breathing 1) in the prehospital setting or on hospital arrival, or 2) both in the prehospital setting or on hospital arrival.

### 2.5 Outcomes

The primary outcome was favorable neurologic outcomes at 30 days. Favorable neurologic outcomes were defined using the Cerebral Performance Category (CPC) scale, which defines neurologic function as follows: CPC1 represents good cerebral performance, CPC2 represents moderate disability, CPC3 represents severe disability, CPC4 indicates coma or vegetative state, and CPC5 indicates brain death or death. CPC1 and CPC2 were defined as favorable outcomes. The secondary outcome was 30-day survival.

### 2.6 Analysis

We summarized patient characteristics using descriptive statistics. We presented continuous variables as median with IQR, whereas categoric variables were presented as counts and percentages. We imputed missing data using the “missForest” package in R, which uses a random forest algorithm. This approach performs well with mixed data types and complex, nonlinear interactions and has shown reasonable accuracy even under partially nonrandom missingness.^21^, ^22^, ^23^ Because “missForest” produces a single completed data set, all primary and sensitivity analyses were conducted using the imputed data set without pooling across multiple imputations. Participants were categorized into 3 groups based on agonal breathing: (1) no agonal breathing, (2) prehospital or hospital agonal breathing, or (3) prehospital and hospital agonal breathing. Associations between agonal breathing and outcomes were examined using multilevel multivariable logistic regression models with random intercepts for facility, with the no agonal breathing group as the reference. Adjusted variables were age, sex, cardiogenic cause, initial shockable rhythm, witness, bystander CPR, public automated external defibrillator (AED), ROSC on transport, advanced airway, prehospital physician intervention, and call-to-hospital arrival time. We performed diagnostic checks to assess multicollinearity among covariates, potential effect measure modification, and the functional form of continuous variables in the multivariable model. Odds ratios (ORs) and 95% CIs were calculated to examine the association between agonal breathing types and the outcomes.

To assess the robustness and consistency of our findings, we conducted 3 prespecified sensitivity analyses. First, to minimize potential misclassification of spontaneous breathing as agonal breathing, we conducted the analysis excluding patients who achieved ROSC at either EMS or hospital arrival. Second, to evaluate the impact of missing data and assess whether random forest imputation influenced the results, we performed a complete-case multivariable logistic regression analysis. Third, given the distinct pathophysiology, we conducted the analysis using the imputed data set while excluding OHCA cases attributed to trauma to ensure internal consistency.

A significance level of *P* < .05 was considered statistically significant. All analyses were performed using R (version 4.4.2, R Foundation for Statistical Computing).

## 3 Results

### 3.1 Patient Characteristics

Between 2019 and 2021, the database recorded data from 9909 patients with OHCA. The median age was 76 (IQR, 62-84) years, 61.6% were male, 37.9% were witnessed, 41.5% received bystander CPR, and 7.7% had an initial shockable rhythm (Table 1).

**Table:** Patient characteristics by agonal breathing type.

Variable	No agonal breathing N = 8956	Prehospital or hospital agonal breathing N = 860	Prehospital and hospital agonal breathing N = 93
Demographic characteristic			
Age, y, median (IQR)	76 (62-84)	75 (63-84)	70 (57.5-81)
Sex, male, n (%)	5474 (61.1)	566 (65.8)	66 (71.0)
Cardiogenic cause, n (%)	5444 (60.8)	519 (60.3)	55 (59.1)
Initial rhythm, n (%)			
Ventricular fibrillation	561 (6.3)	139 (16.2)	28 (30.1)
Pulseless ventricular tachycardia	25 (0.3)	9 (1.0)	1 (1.1)
PEA	1866 (20.8)	364 (42.3)	35 (37.6)
Asystole	6063 (67.7)	138 (16.0)	8 (8.6)
ROSC on EMS arrival	441 (4.9)	210 (24.4)	21 (22.6)
Prehospital information, n (%)			
Witness	3233 (36.0)	472 (54.9)	51 (54.8)
Bystander CPR	3665 (40.9)	396 (46.0)	48 (51.6)
Public AED	140 (1.6)	66 (7.7)	4 (4.3)
ROSC on transport	727 (8.1)	204 (23.7)	33 (35.5)
Advanced airway, n (%)	3974 (44.4)	335 (39.0)	28 (30.1)
Adrenaline, n (%)	2651 (29.6)	239 (27.8)	23 (24.7)
Prehospital physician intervene	608 (6.8)	103 (12.0)	7 (7.5)
Rhythm on arrival, n (%)			
Ventricular fibrillation	281 (3.1)	62 (7.2)	10 (10.8)
Pulseless ventricular tachycardia	26 (0.3)	5 (0.6)	4 (4.3)
PEA	1569 (17.5)	285 (33.1)	24 (25.8)
Asystole	6348 (70.9)	288 (33.5)	22 (23.7)
ROSC on arrival	732 (8.2)	220 (25.6)	33 (35.5)
Time course, min, median (IQR)			
Call to EMS arrival	7 (6-9)	7 (5.75-9)	7 (5-8)
Call-to-hospital arrival	35 (29-42)	35 (27-43)	33 (25.5-40.5)
In-hospital treatment			
Adrenaline, n (%)	6622 (74.0)	577 (67.1)	51 (54.8)
ECPR, n (%)	250 (2.8)	79 (9.2)	12 (12.9)

AED, automated external defibrillator; ECPR, extracorporeal cardiopulmonary resuscitation; EMS, emergency medical services; IQR, interquartile range; PEA, pulseless electrical activity; ROSC, return of spontaneous circulation.

**Figure 1:**
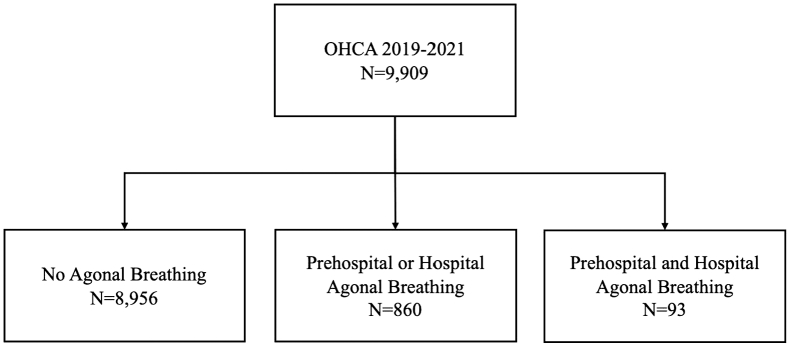
Patients included in the analysis. Patients were categorized into 3 groups according to agonal breathing: (1) no agonal breathing, (2) prehospital or hospital agonal breathing, and (3) prehospital and hospital agonal breathing. No patients were excluded from the analysis. OHCA, out-of-hospital cardiac arrest.

The occurrence of agonal breathing was as follows: prehospital only, n = 750 (7.6%); hospital only, n = 110 (1.1%); and both prehospital and hospital, n = 93 (0.9%) (Fig 1). Information was missing for prehospital agonal breathing in 2806 (28.3%) and for hospital agonal breathing in 2720 (27.4%). Before imputation, characteristics and outcomes by agonal breathing type are summarized in Table S1. Patients with both prehospital and hospital agonal breathing were younger (70 [IQR, 57.5-81.0] years) and more likely to be male (71.0%). The proportions of initial shockable rhythm, witnessed arrest, bystander CPR, and public AED use were higher in patients with agonal breathing. ROSC on hospital arrival was observed in 8.2% of patients without agonal breathing, 25.6% of those with prehospital or hospital agonal breathing, and 35.5% of those with prehospital and hospital agonal breathing.

### 3.2 Main Results

**Figure 2:**
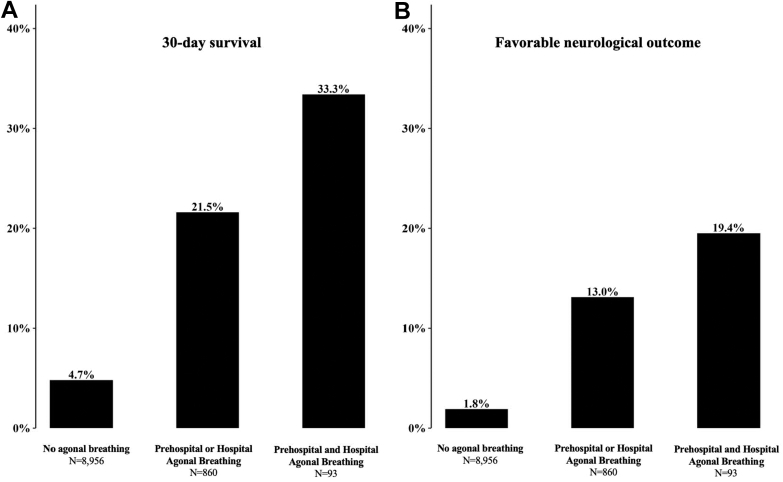
Favorable neurologic outcome and 30-day survival by agonal breathing type. A 30-day survival (A) and a favorable neurologic outcome (B) by agonal breathing category. A favorable neurologic outcome was defined as a Cerebral Performance Category score of 1 or 2.

The outcomes of overall patients showed that favorable neurologic outcomes and 30-day survival were observed in 3.0% and 6.4%, respectively (Fig 2). Favorable neurologic outcomes were observed in 1.8% of the no agonal breathing group, 13.0% of those with prehospital or hospital agonal breathing, and 19.4% of those with both prehospital and hospital agonal breathing. The 30-day survival rates were 4.7%, 21.5%, and 33.3%, respectively. The associations persisted among patients with prehospital or hospital agonal breathing (Table S2).

**Figure 3:**
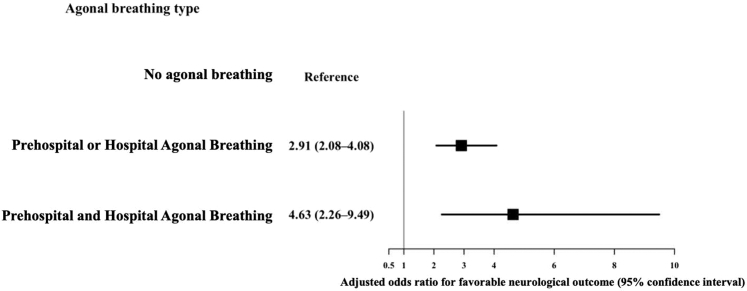
Multivariable logistic regression for a favorable neurologic outcome by agonal breathing type. Models were adjusted for age, sex, cardiogenic cause, initial shockable rhythm, witnessed arrest, bystander cardiopulmonary resuscitation, public automated external defibrillator use, return of spontaneous circulation during transport, advanced airway management, prehospital physician intervention, and call-to-hospital arrival time, with random intercepts for facility.

**Figure 4:**
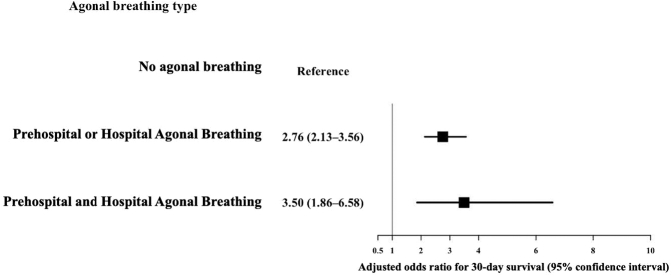
Multivariable logistic regression for 30-day survival by agonal breathing type. Models were adjusted for age, sex, cardiogenic cause, initial shockable rhythm, witnessed arrest, bystander cardiopulmonary resuscitation, public automated external defibrillator use, return of spontaneous circulation during transport, advanced airway management, prehospital physician intervention, and call-to-hospital arrival time, with random intercepts for facility.

Multilevel multivariable logistic regression analysis with random intercepts for facility demonstrated that, compared with the no agonal breathing group, either or both prehospital and hospital agonal breathing had higher odds of a favorable neurologic outcome ([OR, 2.91; 95% CI, 2.08-4.08] and [OR, 4.63; 95% CI, 2.26-9.49]) (Fig. 3; full model shown in Table S3). Agonal breathing was similarly associated with higher 30-day survival (Fig. 4).

### 3.3 Sensitivity Analysis

Across all 3 prespecified sensitivity analyses, the direction and magnitude of associations were similar to the primary analysis. First, excluding patients who achieved ROSC either in the prehospital setting or on hospital arrival, prehospital or hospital agonal breathing was associated with a favorable neurologic outcome (OR, 5.62; 95% CI, 2.58-12.22), as was prehospital and hospital agonal breathing (OR, 16.36; 95% CI, 4.70-56.92) (Table S4). Second, in the complete-case multilevel analysis, prehospital or hospital agonal breathing (OR, 2.38; 95% CI, 1.57-3.59) and prehospital and hospital agonal breathing (OR, 5.21; 95% CI, 2.25-12.04) remained associated with a favorable neurologic outcome (Table S5). Third, excluding OHCA cases attributed to trauma, similar associations were observed for prehospital or hospital agonal breathing (OR, 3.40; 95% CI, 2.45-4.73) and for prehospital and hospital agonal breathing (OR, 3.88; 95% CI, 1.95-7.75) (Table S6).

## 4 Limitations

Several limitations should be considered. First, the definition and detection of agonal breathing are inherently challenging. Agonal breathing lacks standardized criteria for duration or frequency, and assessments rely on clinical judgment that varies among health care workers. Our 2-time-point classification approximates persistence, but misclassification remains possible. A sensitivity analysis excluding patients with ROSC at either EMS or hospital arrival yielded consistent results. Future studies should incorporate more objective methods, such as impedance-based monitoring, to improve reproducibility.^24^ Second, approximately 30% of the data on agonal breathing were missing in this study. These missing data were mainly due to incomplete documentation in this prospective registry rather than systematic bias. We applied multiple imputation using the random forest algorithm. Although random-forest-based imputations may be affected by nonrandom missingness, data skewness, and high missing proportions, recent evidence suggests that this approach performs robustly even in complex, nonlinear data sets.^21^, ^22^, ^23^ In this study, the distributions and associations of key variables were similar between the imputed and non-imputed datasets, indicating that the main findings were largely robust to missingness, although some residual uncertainty cannot be ruled out. Third, the overall incidence of a favorable neurologic outcome and survival was low, and the number of patients with prehospital and hospital agonal breathing was limited. Therefore, the statistical precision of the logistic regression estimates may be reduced, and the findings should be interpreted with caution. Fourth, this study was conducted in a cohort from emergency centers located in the metropolitan area of Japan, which may introduce selection bias. However, when compared to nationwide registry analyses and global data,^25^^,^^26^ there were no significant deviations in patient background, bystander CPR, AED use, EMS arrival times, or hospital arrival times. Fifth, heterogeneity within the OHCA population may influence interpretation. Although prior studies suggest similar prognostic patterns in pediatric and traumatic OHCA,^16^^,^^17^ physiological differences across these subgroups could still modify the association. In the present study, a subgroup analysis excluding traumatic OHCA demonstrated similar tendencies, supporting the robustness of the main findings. Finally, given the observational design, causal inferences cannot be made, and the observed relationships should be interpreted as associations.

## 5 Discussion

In this study, we described the course and outcomes of patients exhibiting agonal breathing among 9909 patients with OHCA. Agonal breathing was associated with a higher likelihood of favorable neurologic outcomes, with patients showing both prehospital and hospital agonal breathing exhibiting the highest odds of a favorable outcome. The associations persisted even among those with only prehospital or hospital agonal breathing.

Previous studies have reported that agonal breathing is associated with favorable outcomes, but these analyses mainly evaluated agonal breathing only in the prehospital or hospital phases of OHCA care. A representative multicenter study by Debaty et al^5^ demonstrated that gasping during cardiopulmonary resuscitation was independently associated with higher 1-year survival with a favorable neurologic outcome, and similar associations between prehospital agonal breathing and higher survival, sustained ROSC, and favorable neurologic outcomes have been reported in other observational studies.^6^^,^^8^^,^^9^^,^^27^ Studies by Kitano et al^12^ show that agonal breathing at hospital arrival is significantly associated with more favorable neurologic outcomes. In a study focusing on patients who underwent ECPR, 8.0% of patients exhibited agonal breathing at hospital arrival, which was associated with more favorable neurologic outcomes.^13^ Furthermore, the only study that observed multiple time points reported that agonal breathing was associated with increased favorable outcomes, particularly when it was continuous during transport to the hospital. However, this study was limited to 212 cases, all of which had shown an initial shockable rhythm and received ECPR.^28^ Our study differs from the prior efforts by examining the joint associations of both prehospital and hospital agonal breathing.

Prehospital and hospital arrival agonal breathing were thought to have different clinical and physiological implications. First, prehospital agonal breathing could indicate a shorter no-flow time. The study from the Czech Republic reported that 33% of patients who had cardiac arrest after EMS contact exhibited agonal breathing, and among witnessed patients with OHCA, the prevalence of agonal breathing at EMS arrival was 20% when EMS arrival time was less than 7 minutes, 14% when between 7 and 9 minutes, and 7% when >9 minutes.^29^ Furthermore, the study found that agonal breathing at EMS arrival was significantly associated with a shorter response time.^6^ In a study of patients who received ECPR, the presence of agonal breathing was associated with both bystander CPR and shorter no-flow time.^30^

On the other hand, agonal breathing at hospital arrival could suggest adequate cerebral blood flow during transport.^12^^,^^13^ A previous animal study has shown that when CPR is inadequate, agonal breathing rapidly diminishes,^31^ which supports the idea that agonal breathing at hospital arrival could serve as an indicator of effective CPR during transport. Patients who exhibited agonal breathing at both time points likely received early CPR after cardiac arrest and continued to receive effective chest compressions that maintained cerebral blood flow. This finding supports that this group had the highest probability of a favorable outcome.

This study provides epidemiological data on agonal breathing observed at both EMS contact and hospital arrival and demonstrates its significance as a prognostic factor. This study highlights the prognostic value of the temporal pattern of agonal breathing and provides a foundation for incorporating it into future prognostic assessment frameworks. Taken together, the subjectivity in defining and recording agonal breathing underscores the need for clearer definitions, systematic observation, and objective assessment. Building on our findings, future research should incorporate agonal breathing into prognostic and risk-stratification frameworks, where it may serve as a clinically meaningful indicator associated with neurologic outcomes. Further work is needed to clarify its relevance across different OHCA subgroups.

In conclusion, the presence of agonal breathing was associated with a higher likelihood of a favorable neurologic outcome, particularly when agonal breathing was observed both in the prehospital setting and on hospital arrival. Although a definitive definition of agonal breathing remains lacking, collecting information on the duration of agonal breathing may be important in future studies.

## Author Contributions

Hiroaki Taniguchi: Writing – original draft, Visualization, Methodology, Investigation, Formal analysis, Conceptualization. Kohei Yamada: Writing – review & editing, Methodology. Shinnosuke Kitano: Writing – review & editing, Methodology. Naofumi Bunya: Writing – review & editing, Methodology. Yosuke Homma: Writing – review & editing, Project administration, Data curation, Conceptualization. Takashi Tagami: Writing – review & editing, Project administration, Data curation, Conceptualization. Makoto Aoki: Writing – review & editing, Project administration, Data curation, Formal analysis, Conceptualization.

## Funding and Support

Naofumi Bunya reports receiving research funding from the Japan Society for the Promotion of Science KAKENHI (Grant Number: 23K08480) for studies related to signs of life in cardiac arrest. Makoto Aoki reports receiving research funding from the Japanese Society for the Promotion of Science KAKENHI (Grant Number: 24K23501). Authors declares no other conflicts of interest relevant to this article.

## Conflict of Interest

The authors declare no competing interests.
