# Supplementary material for: Association between agonal breathing and outcomes after out-of-hospital cardiac arrest: a retrospective study
Source: J Am Coll Emerg Physicians Open. 2026 Mar 7;7(2):100345. doi: 10.1016/j.acepjo.2026.100345 (PMC12991942; doi:10.1016/j.acepjo.2026.100345)
Supplement: Supplementary Table 1-6 [file mmc1.docx]

**Association between agonal breathing and outcomes after out-of-hospital cardiac arrest: a retrospective study**

**Supplementary Appendix**

**Table of contents.**

**Tables**

**Table S1. Characteristics and outcomes of study patients by agonal breathing type without imputation.**

……………...Page 2-3

**Table S2. Characteristics and outcomes of patients with only prehospital or hospital agonal breathing.**

………………...Page 4

**Table S3**. **Full multilevel logistic regression models for favorable neurological outcome**

………………...Page 5-6

**Table S4. Sensitivity analysis of adjusted outcomes by agonal breathing type, excluding patients who had ROSC on EMS arrival or hospital arrival.**

………………...Page 7

**Table S5. Sensitivity analysis of adjusted outcomes by agonal breathing type, using the complete-case dataset**

………………...Page 8

**Table S6. Sensitivity analysis of adjusted outcomes by agonal breathing type, excluding traumatic causes.**

………………...Page 9

**Table S1.** **Characteristics and outcomes of study patients by agonal breathing type without imputation.**

| Variable | No agonal breathing  n= 5,197 | Prehospital or Hospital Agonal Breathing  n= 539 | Prehospital and Hospital Agonal Breathing  n= 62 |
| --- | --- | --- | --- |
| Demographics  Age, years, median (IQR)  Sex, male, n (%)  Cardiogenic cause, n (%)  Non-cardiogenic cause, n (%)  Cerebrovascular disease  Respiratory disease  Trauma  Other | 76 (62-84)  3199 (61.6)  3171 (61.0)  130 (2.5)  237 (4.6)  301 (5.8)  1358 (26.1) | 75 (60-84)  353 (65.5)  330 (61.2)  23 (4.3)  29 (5.4)  24 (4.5)  133 (24.7) | 71 (56-82)  46 (74.2)  41 (66.1)  1 (1.6)  6 (9.7)  1 (1.6)  13 (21.0) |
| Initial rhythm, n (%)  Ventricular fibrillation  Pulseless ventricular tachycardia  PEA  Asystole  ROSC on EMS arrival | 309 (5.0)  12 (0.2)  1,035 (19.9)  3,432 (66.0)  265 (5.1) | 87 (16.1)  7 (1.3)  220 (40.8)  103 (19.1)  112 (20.8) | 18 (29.0)  1 (1.6)  24 (38.7)  7 (11.3)  10 (16.1) |
| Prehospital information, n (%)  Witness  Bystander CPR  Public AED  ROSC on transport  Advanced airway, n (%)  Adrenaline, n (%)  Prehospital physician intervene | 1,821 (35.0)  2,170 (41.8)  80 (1.5)  424 (8.2)  2,437 (46.9)  1,568 (30.2)  358 (6.9) | 302 (56.0)  253 (46.9)  37 (6.9)  130 (24.1)  241 (44.7)  174 (32.3)  69 (12.8) | 34 (54.8)  31 (50.0)  3 (4.8)  16 (25.8)  23 (37.1)  18 (29.0)  5 (8.1) |
| Rhythm on arrival, n (%)  Ventricular fibrillation  Pulseless ventricular tachycardia  PEA  Asystole  ROSC on arrival | 154 (3.0)  17 (0.3)  853 (16.4)  3,760 (72.3)  413 (7.9) | 41 (7.6)  2 (0.4)  162 (30.1)  199 (36.9)  135 (25.0) | 10 (16.1)  1 (1.6)  19 (30.6)  17 (27.4)  15 (24.2) |
| Time course, min, median (IQR)  Call to EMS arrival  Call to hospital arrival | 8 (6-10)  33 (27-40) | 7 (6-9)  33 (27-42) | 8 (6-9)  27 (21.5-36.5) |
| In-hospital treatment  Adrenaline, n (%)  ECPR, n (%) | 3654 (70.3)  132 (2.5) | 351(65.1)  48 (8.9) | 40 (64.5)  10 (16.1) |
| Outcomes, n (%)  30day survival  Favorable neurologic outcome | 225 (4.3)  104 (2.0) | 98 (18.2)  63 (11.7) | 18 (29.0)  14 (22.6) |

IQR, interquartile range; PEA, pulseless electrical activity; ROSC, return of spontaneous circulation; EMS, emergency medical services; AED, automated external defibrillator; ECPR, extracorporeal cardiopulmonary resuscitation.

**Table S2. Characteristics and outcomes of patients with only prehospital or hospital agonal breathing.**

| Variable | With agonal  Hospital arrival  (n = 110) | With agonal  EMS arrival  (n = 750) |
| --- | --- | --- |
| Demographics  Age, years, median (IQR)  Sex, male, n (%)  Cardiogenic cause, n (%) | 78 (67-84)  70 (63.6)  63 (57.2) | 74 (62-84)  496 (66.1)  456 (60.8) |
| Initial rhythm, n (%)  Ventricular fibrillation  Pulseless ventricular tachycardia  PEA  Asystole  ROSC on EMS arrival | 15 (13.6)  0 (0)  30 (27.3)  48 (43.6)  17 (15.5) | 124 (16.5)  9 (1.2)  334 (44.5)  90 (12.0)  193 (25.7) |
| Prehospital information, n (%)  Witness  Bystander CPR  Public AED  ROSC on transport  Advanced airway, n (%)  Adrenaline, n (%)  Prehospital physician intervene | 51 (46.3)  53 (48.1)  8 (7.3)  32 (29.0)  56 (50.9)  47 (42.7)  9 (8.2) | 421 (56.1)  343 (45.7)  58 (7.7)  172 (22.9)  279 (37.2)  192 (25.6)  94 (12.5) |
| Rhythm on arrival, n (%)  Ventricular fibrillation  Pulseless ventricular tachycardia  PEA  Asystole  ROSC on arrival | 10 (9.1)  1 (0.9)  25 (22.7)  36 (32.7)  38 (34.5) | 52 (6.9)  4 (0.5)  260 (34.7)  252 (33.6)  182 (24.3) |
| Time course, min, median (IQR)  Call to EMS arrival  Call to hospital arrival | 6.5 (5- 9)  34 (28.75-41) | 7 (6-8)  35 (27-43) |
| In-hospital treatment  Adrenaline, n (%)  ECPR, n (%) | 67 (60.9)  6 (5.5) | 510 (68.0)  73 (9.7) |
| Outcomes, n (%)  30day survival  Favorable neurologic outcome | 21 (19.1)  11 (10.0) | 164 (21.9)  101 (13.5) |

IQR, interquartile range; PEA, pulseless electrical activity; ROSC, return of spontaneous circulation; EMS, emergency medical services; AED, automated external defibrillator; ECPR, extracorporeal cardiopulmonary resuscitation.

**Table S3. Full multilevel logistic regression models for favorable neurological outcome**

| Variable | Imputed dataset |
| --- | --- |
| No agonal breathing | Reference |
| Prehospital or Hospital Agonal Breathing | 2.91 (2.08-4.08) |
| Prehospital and Hospital Agonal Breathing | 4.63 (2.26-9.49) |
| Age, male | 0.99 (0.98–0.99) |
| Sex, male | 1.45 (1.03–2.04) |
| Cardiogenic cause | 2.65 (1.82–3.87) |
| Initial shockable rhythm | 3.32 (2.34–4.73) |
| Witness | 1.49 (1.09–2.03) |
| Bystander | 1.57 (1.15–2.14) |
| Public AED | 7.08 (4.43–11.31) |
| ROSC on transport | 13.91 (10.27–18.83) |
| Advanced airway, | 0.22 (0.16–0.32) |
| Prehospital physician intervene | 0.78 (0.47–1.31) |
| Call to hospital arrival | 0.99 (0.98–1.00) |

**AED, automated external defibrillator; ROSC, return of spontaneous circulation. All values are shown as adjusted odds ratios (95% confidence intervals).**

**Table S4. Sensitivity analysis of adjusted outcomes by agonal breathing type, excluding patients who had ROSC on EMS arrival or hospital arrival.**

| Outcome | Group | aOR (95%CI) |
| --- | --- | --- |
| Favorable neurological outcome | No agonal breathing  Prehospital or Hospital Agonal Breathing  Prehospital and Hospital Agonal Breathing | Reference  5.62 (2.58-12.22)  16.36 (4.70-56.92) |
| Survival | No agonal breathing  Prehospital or Hospital Agonal Breathing  Prehospital and Hospital Agonal Breathing | Refence  2.59 (1.60-4.17)  6.68 (2.60-17.18) |

ROSC, return of spontaneous circulation; EMS, emergency medical services; aOR, adjusted odds ratio; CI, confidence interval

Models were adjusted for age, sex, cardiogenic cause, initial shockable rhythm, witnessed arrest, bystander cardiopulmonary resuscitation, public automated external defibrillator use, return of spontaneous circulation during transport, advanced airway management, prehospital physician intervention, and call-to-hospital arrival time, with random intercepts for facility.

**Table S5. Sensitivity analysis of adjusted outcomes by agonal breathing type, using the complete-case dataset**

| Outcome | Group | aOR (95%CI) |
| --- | --- | --- |
| Favorable neurological outcome | No agonal breathing  Prehospital or Hospital Agonal Breathing  Prehospital and Hospital Agonal Breathing | Reference  2.38 (1.57–3.59)  5.21 (2.25–12.04) |
| Survival | No agonal breathing  Prehospital or Hospital Agonal Breathing  Prehospital and Hospital Agonal Breathing | Refence  2.35 (1.70-3.24)  4.00 (1.91-8.39) |

Abbreviations: aOR, adjusted odds ratio; CI, confidence interval.

Models were adjusted for age, sex, cardiogenic cause, initial shockable rhythm, witnessed arrest, bystander cardiopulmonary resuscitation, public automated external defibrillator use, return of spontaneous circulation during transport, advanced airway management, prehospital physician intervention, and call-to-hospital arrival time, with random intercepts for facility.

**Table S6. Sensitivity analysis of adjusted outcomes by agonal breathing type, excluding traumatic causes.**

| Outcome | Group | aOR (95%CI) |
| --- | --- | --- |
| Favorable neurological outcome | No agonal breathing  Prehospital or Hospital Agonal Breathing  Prehospital and Hospital Agonal Breathing | Reference  3.40 (2.45-4.73)  3.88 (1.95-7.75) |
| Survival | No agonal breathing  Prehospital or Hospital Agonal Breathing  Prehospital and Hospital Agonal Breathing | Refence  3.06 (2.37-3.94)  3.77 (2.10-6.78) |

Abbreviations: aOR, adjusted odds ratio; CI, confidence interval.

Models were adjusted for age, sex, cardiogenic cause, initial shockable rhythm, witnessed arrest, bystander cardiopulmonary resuscitation, public automated external defibrillator use, return of spontaneous circulation during transport, advanced airway management, prehospital physician intervention, and call-to-hospital arrival time, with random intercepts for facility.
